# Behavioural Responses of *Unio tumidus* Freshwater Mussels to Pesticide Contamination

**DOI:** 10.1007/s00244-019-00649-2

**Published:** 2019-07-04

**Authors:** Joanna Chmist, Krzysztof Szoszkiewicz, Dariusz Drożdżyński

**Affiliations:** 1 Department of Ecology and Environmental Protection, Faculty of Environmental Engineering and Spatial Management, Poznań University of Life Sciences, Wojska Polskiego 28 street, 60-637 Poznań, Poland; 2 Institute of Plant Protection in Poznań, Węgorka 20 Street, 60-318 Poznań, Poland

## Abstract

A pesticide is a chemical substance used for the disposal of pests, such as insects, weeds, invertebrates, or rodents. Pesticides interfere with the normal metabolism of the target species; however, some of them may inadvertently affect organisms other than those targeted. Increased quantities of pesticides in water disturb various ecological processes and may increase the mortality rate of various native species of flora and fauna. One of the groups of organisms that are at the greatest risk from the adverse effects of pesticides is the bivalves. This study was designed to assess the behavioural reaction of bivalves to widespread pesticides. As a representative example, the Polish native *Unio tumidus* (Philipsson 1788) was used. The study investigated different groups of toxic pesticides, such as herbicides (lenacil), insecticides (thiacloprid, DDT and dichlorvos), and fungicides (tebuconazole), in concentrations of 10 mg L^−1^. The results showed various behavioural reactions of bivalves to the pesticides. The most evident were activity time and shell opening rate. Moreover, as a result of DDVP contamination, effects were recorded in terms of shell opening level as well as rapid onset of death. Among the five analysed plant protection products, the most toxic was DDVP. Its presence caused adductor muscle paralysis in all analysed individuals. The least toxic pesticides were DDT and thiacloprid. A strong reaction to lenacil was observed especially in the shell opening rate. Tebuconazole caused significant reductions in activity. Despite the fact that the impact of pesticides on ecosystems is under regular observation, with the use of a wide range of scientific techniques, the use of bivalves was shown to have considerable potential for water quality monitoring.

Pesticides are among the main stressors in aquatic ecosystems located in agricultural areas (Pathiratne and Kroon 2016). Due to farming, every year more than one million tonnes of fertilizers and pesticides contaminate both surface and groundwater (Cruzeiro et al. 2016; Rodrigues et al. 2018). Increased quantities of pesticides in water disturb various ecological processes and may increase the mortality rate of various native species of flora and fauna (Edwards 2013; Nowell et al. 2014; Serrano et al. 2012).

Levels of pesticides in the environment are increasing year by year. Until the Second World War, only 30 pesticides were known. However, the peak quantity of pesticides used worldwide (more than 1.8 billion kilograms per year) was recorded between 1960 and 1980 (Renault 2011). Currently, more than 1700 plant protection products, containing more than 220 active substances, are registered and used in Poland. However, due to the withdrawal from the market of some groups of pesticides in the 1970s, their excess was deposited in concrete wells. According to estimates of the Ministry of Environmental Protection, the total weight of pesticide waste in Poland may be as high as 60,000 tonnes. Data obtained by Ignatowicz (2007) show that the technical condition of these wells is very poor, leading to the emission of toxins and poisons to the natural environment. It has been shown that the concentration of several pesticides in water ecosystems is 100 times higher than that specified in the current regulations of the Minister of Health (2017) on the quality of water intended for human consumption (Niewiadowska et al. 2014).

The test pesticides were selected based on their wide use in Poland (or elsewhere) at present and in the past (Kucharski et al. 2011; Roszko et al. 2016). The study considered different groups of pesticides, such as herbicides, insecticides, and fungicides. The selected herbicide was a uracil substance called lenacil (3-cyclohexyl-1,5,6,7-tetrahydrocyclopentapyrimidine-2,4(3*H*)-dione), which in Poland and other countries is widely used in agriculture to control weeds. Lenacil is the active substance in many herbicide products. Lenacil is absorbed by the roots and translocated throughout the plant (Kucharski et al. 2011). The group of insecticides was represented by thiacloprid ((Z)-3-(6-chloro-3-pyridylmethyl)-1,3-thiazolidin-2-ylidenecyanamide), dichlorodiphenyltrichloroethane (1,1,1-trichloro-2,2-bis(4-chlorophenyl)ethane), and dichlorvos (2,2-dichlorovinyl dimethyl phosphate). Thiacloprid is an insecticide of the neonicotinoid class. The mechanism of its action is similar to that of other insecticides from the neonicotinoids group; it disturbs the insect’s nervous system by stimulating nicotinic acetylcholine receptors (Schuld and Schmuck 2000). Dichlorodiphenyltrichloroethane, commonly known as DDT, is a compound that was developed as an organochlorine insecticide/acaricide. DDT has been shown to cause a number of adverse effects in animals. The side-effects of this insecticide mainly involve disorders of the reproductive, neurological, and immunological systems of animals (Bian et al. 2009). Dichlorvos, commonly known as DDVP, was widely used as an organophosphate insecticide/acaricide but was banned in December 2008. This compound gained notoriety due to its frequent occurrence in urban waterways. Despite its insecticidal purpose, its toxicity extends beyond insects. It is toxic to both invertebrates and vertebrates, including humans (Bolton-Warberg et al. 2007). Among the fungicides, tebuconazole was selected. Tebuconazole ((RS)-1-*p*-chlorophenyl-4,4-dimethyl-3-(1*H*-1,2,4-triazol-1-ylmethyl)pentan-3-ol) is a very popular conazole fungicide (from the triazole fungicides group) used in agriculture to treat plant pathogenic fungi. It is classified as toxic to aquatic organisms. It may cause long-term adverse effects in the aquatic ecosystem (Yu et al. 2013). Tebuconazole is one of the most frequently detected pesticides in surface waters in Wielkopolska province, both lentic and lotic. In the years 2015–2017, tebuconazole was detected in 35.7%, 25.5%, and 52.8% (respectively) of all water samples collected and analysed at the Institute of Plant Protection NRI in Poznań, Poland. Tebuconazole, thiacloprid, and lenacil are still registered and widely used in Polish agriculture (Roszko et al. 2016).

Increasing the application of pesticides requires caution in use and more studies on their impact on ecosystems (Falconer 2006; Kazi et al. 2009). One of the groups of organisms that are at the greatest risk from the adverse effects of pesticides is the bivalves—one of the most varied and species-rich groups of aquatic creatures (Oehlmann and Schulte-Oehlmann 2003; Bouilly et al. 2007). Bivalves play an important ecological role in various ecosystems, both freshwater and marine, mainly because they are efficient water filters. In addition, they are good indicators of environmental quality, especially for rivers and lakes (Donrovich et al. 2017). Due to their relatively sedentary lifestyle, they have limited abilities to escape in the event of threats (Cossu et al. 2000).

The effects of pesticides on bivalves have already been studied in various types of ecosystems, although the number of publications is still very limited (Galloway et al. 2002; Greco et al. 2010). This study provided a unique outlook on the impact of pesticides on bivalves. Using original laboratory equipment, highly precise monitoring of various elements of the bivalves’ behaviour was possible. The testing of five types of pesticides of particular significance for the environment aligns with the challenges faced in contemporary agricultural ecosystems. We hypothesized that pesticide contamination has a significant impact on bivalves, reflected by various behavioural reactions, such as activity time, shell opening rate, and shell opening level.

## Materials and Methods

### Tested Pesticides

This study focused on toxic pesticides which pose a serious risk to the environment. According to the Globally Harmonized System of Classification and Labelling of Chemicals (GHS), all of them are classified as H400: very toxic to aquatic life, or as H410: very toxic to aquatic life with long lasting effects (Table 1).

**Table 1:** Characteristics of the five pesticides tested according to according to the Globally Harmonized System of Classification and Labelling of Chemicals (GHS)

Pesticide	Agrochemical category	Molecular formula	Hazards identification (GHS)	Reference
Lenacil	Herbicide	C_13_H_18_N_2_O_2_	H351: Suspected of causing cancer	European Chemicals Agency (ECHA)
			H400: Very toxic to aquatic life	
			H410: Very toxic to aquatic life with long-lasting effects	
DDT	Insecticide	C_14_H_9_C_l5_	H301 + H311: Toxic if swallowed or in contact with skin	European Chemicals Agency (ECHA)
			H301: Toxic if swallowed	
			H311: Toxic in contact with skin	
			H351: Suspected of causing cancer	
			H372: Causes damage to organs through prolonged or repeated exposure	
			H400: Very toxic to aquatic life	
			H410: Very toxic to aquatic life with long-lasting effects	
Thiacloprid	Insecticide	C_10_H_9_ClN_4_S	H301: Toxic if swallowed	European Chemicals Agency (ECHA)
			H332: Harmful if inhaled	
			H336: May cause drowsiness or dizziness	
			H351: Suspected of causing cancer	
			H360FD: May damage fertility; May damage the unborn child	
			H400: Very toxic to aquatic life	
			H410: Very toxic to aquatic life with long-lasting effects	
DDVP	Insecticide	C_4_H_7_Cl_2_O_4_P	H301: Toxic if swallowed	EU REGULATION (EC) No 1272/2008
			H311: Toxic in contact with skin	
			H317: May cause an allergic skin reaction	
			H330: Fatal if inhaled	
			H400: Very toxic to aquatic life	
Tebuconazole	Fungicide	C_16_H_22_ClN_3_O	H320: Causes eye irritation	EU REGULATION (EC) No 1272/2008
			H330: Fatal if inhaled	
			H351: Suspected of causing cancer	
			H361: Suspected of damaging fertility or the unborn child	
			H371: May cause damage to organs	
			H373: Causes damage to organs through prolonged or repeated exposure	
			H400: Very toxic to aquatic life	
			H410: Very toxic to aquatic life with long-lasting effects	

In the experiments, each pesticide compound was introduced in a nominal concentration of 10 µg L^−1^. The pesticide concentration was detected in separate experiments with the insecticides, fungicide, and herbicide. A method of solid phase extraction was used for analysis of the changes in concentration (instrumental analysis using liquid chromatography with LC–MS/MS mass detection). The analysis was performed in duplicate, and the results from the replicates were averaged. During the experiment, water samples were analysed three times (24 h, 72 h, and 120 h after exposure).

### *Unio tumidus* Maintenance and Exposure

Freshwater *Unio tumidus* bivalves were collected from a water reservoir in Wielkopolska province, Poland, in 2017 and 2018. All of the bivalves were estimated to be adults with ages ranging from 6 to 9 years (Jakubik and Lewandowski 2016) and were uniform in size (*N* = 40, 6 ± 1-cm length and 3.5 ± 0.5-cm width). All selected bivalves were transported in special tanks courtesy of the Department of Ecology and Environmental Protection laboratory at Poznań University of Life Sciences. They were placed in an aquarium (125 L) with continuous aeration and with access to food. All bivalves were left for approximately 3 weeks to reduce the stress associated with transport. Fine sand was placed at the bottom of the tank. During the experiment, the water pH ranged between 7.6 and 8.4 at a temperature of between 10 and 12 °C, and dissolved oxygen (DO) was maintained at a level between 7.8 and 8.1 mg L^−1^. No mortality was observed during the acclimatization period.

Stock solutions of the test chemicals were prepared in 10 µg L^−1^ concentrations. The selected organisms were randomly divided into five groups of eight individuals. During both the control and exposure periods the bivalves were not fed. The test concentrations were fixed for each test substance based on reference literature (Ignatowicz 2007). The bivalves were exposed for 168 h (including the day of introduction of the substance). Behavioural observations were made for every second, and the median for each day was taken for analysis. For each stock solution, the characteristic behaviour of the bivalves before and after the introduction of the pesticide was noted. The bivalves spent 20 days in the system: 9 days for acclimation, 5 days to establish behaviour under control conditions, and 7 days of exposure to the pesticide. Due to the toxicity of the pesticides, some bivalves died. They were removed from the system to maintain a suitable environment.

### Construction of the Behaviour Monitoring System

**Fig. 1:**
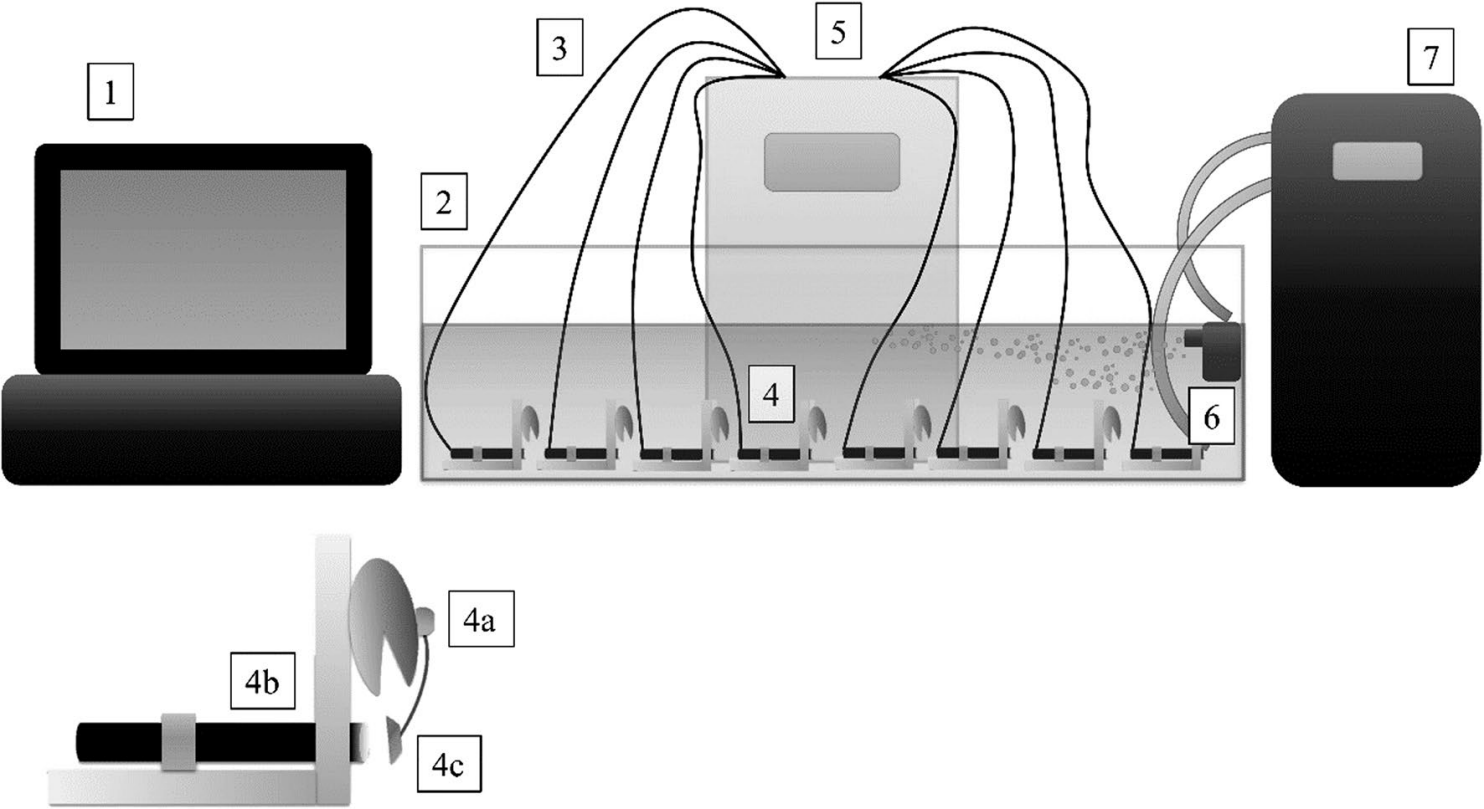
Construction of biological early warning system: (1) computer with dedicated software; (2) aquarium; (3) connection of sensors with the controller; (4) organisms connected to the system; (4a) bivalve; (4b) hall sensor; (4c) magnet; (5) controller; (6) air pump; (7) aquarium chiller

The standard Biological Early Warning System was used in this study; its construction is shown in Fig. 1. The changes in shell movements were measured by a Hall sensor (a transducer for magnetic field strength) with a small ferrite magnet (diameter 8 mm) connected to it. All shell movements were measured continuously and recorded by software.

**Fig. 2:**
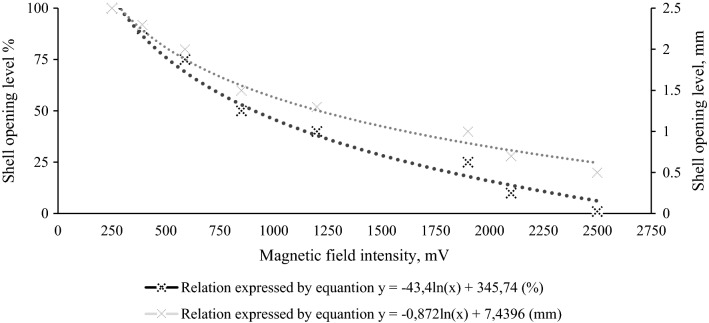
Relationship between the magnetic field intensity and the shell opening level

The system was based on magnetic field changes. Hall sensors were selected as magnetic field transducers due to their small physical dimensions and high sensitivity (Prauzner and Ptak 2014). The magnetic field changes caused by movement of the magnet are converted to the corresponding percentage level of shell opening (Fig. 2). The minimum and maximum range is established for each individual during calibration, in accordance with the relationship shown in Fig. 2.

For each experiment, 8 bivalves were placed in an aquarium (150 × 25 × 25 cm) filled with aerated commercially distributed drinkable spring water (water parameters: HCO_3_^−^ 134.20 mg L^−1^, SO_4_^2−^ 69.54 mg L^−1^, Cl^−^ 18.00 mg L^−1^, F^−^ 0.16 mg L^−1^, Ca^2+^ 60.12 mg L^−1^, Mg^2+^ 13.37 mg L^−1^, Na^+^ 5.00 mg L^−1^, K^+^ 0.75 mg L^−1^, temperature 14–16 °C, DO 7.8–9.1 mg L^−1^, pH 8.0–8.4), circulated using an air pump (Fig. 1). Changes in valve movements were monitored continuously.

Changes in the shell opening level of the exposed bivalves, measured in real time, were compared with the control period. Five days in clean water at a temperature of 10 to 12 ± 1 °C was taken to represent control conditions. In the case of shell opening rate and activity time, before the introduction of the pesticides, the behaviour was very stable and the data were analysed in a block, without any division into individual days. Approximately 86,400 behaviour change records were collected each day.

### Statistics

Statistical differences between the control and the treatment period were tested using the Kruskal–Wallis ANOVA by rank test (*p* < 0.05). Tests for data normality were performed previously.

## Results

### Shell Opening Level

**Fig. 3:**
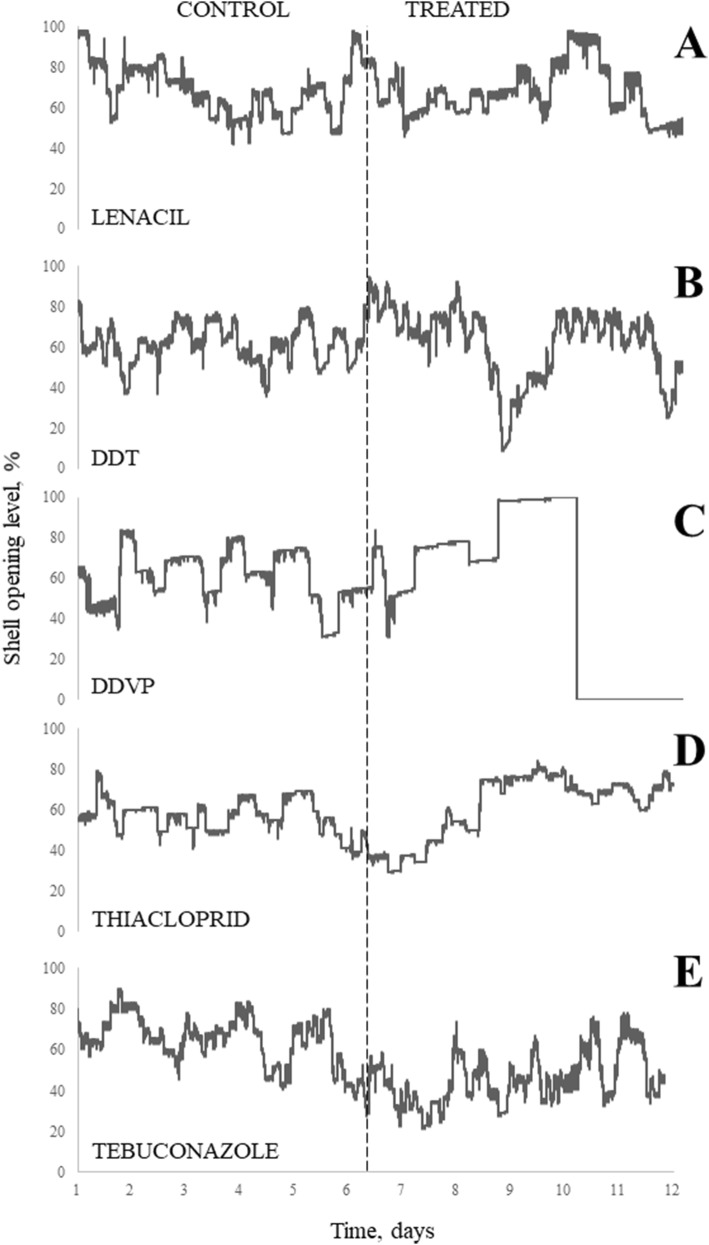
Changes in shell opening level before and during treatment period. **a** Shell opening level for Lenacil, **b** shell opening level for DDT, **c** shell opening level for DDVP, **d** shell opening level for Thiacloprid, **e** shell opening level for Tebuconazole

The application of pesticides induced quite a limited reaction in the shell opening level of the bivalves. The average level (daily median) of shell opening for the set of eight tested bivalves showed fluctuations after a decrease during the first day after exposure (lenacil, thiacloprid, tebuconazole) or after an initial decrease following treatment (DDT and DDVP). The strongest stress reaction was observed in the case of DDVP treatment, where significant differences were detected between the control and treatment periods (*p* > 0.0001, *H* = 54.80). After 8 days, the shell opening level increased to 100%. This was the result of total muscle paralysis, which led to a lethal state. All eight bivalves died during the experiment (Fig. 3c).

### Shell Opening Rate

The application of pesticides caused a clear change in the shell opening rate. During the control period, in each group, the shell opening rate ranged from 0.006 to 0.009 cm s^−1^. The difference results from the greater activity of some bivalves. In the absence of an additional stress factor, the increase in this parameter indicates an increased frequency of shift from activity to rest and vice versa.

**Fig. 4:**
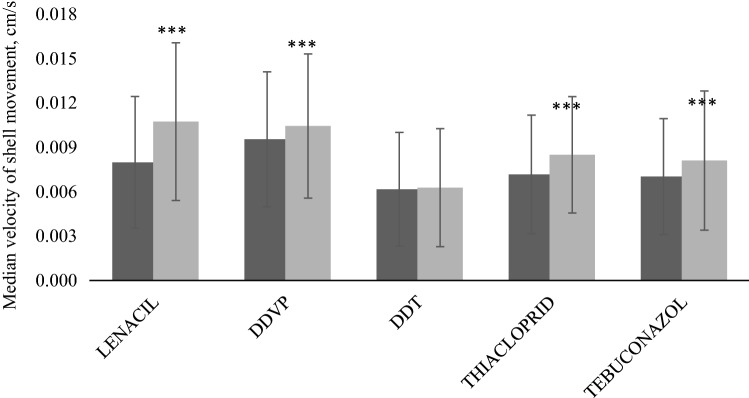
Median shell opening rate before and after exposure to pesticides. Control period (dark colour); treated period (light colour). Significant differences are marked by * where *0.05 < *p* < 0.01, **0.01 ≤ *p *< 0.001, ***p ≤ 0.001

The most apparent increase in shell opening rate was caused by lenacil treatment (median increase by 0.03 cm s^−1^) (Fig. 4). A significant reaction was also detected as a result of treatment with tebuconazole (0.001 cm s^−1^) and with two of the tested insecticides: DDVP (0.002) and thiacloprid (0.002). The impact of all of these pesticides was highly significant according to Kruskal–Wallis ANOVA rank tests with *p* < 0.001. The impact of DDT on the shell opening rate was very limited, and no significance was confirmed (*p* = 0.189).

### Activity Time

The daily activity time during the control period ranged from 10 to 17 h. The differences result from the life cycles of individual specimens.

**Fig. 5:**
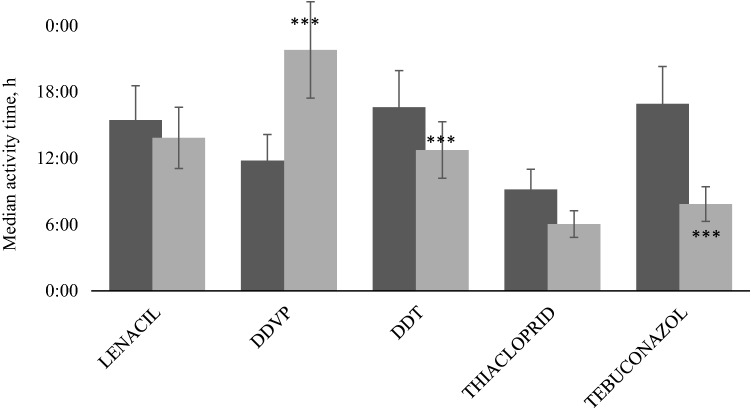
Median activity time before and after exposure to pesticides. Control period (dark colour); treated period (light colour). Significant differences are marked by * where *0.05 < *p* < 0.01, **0.01 ≤ *p *< 0.001, ***p ≤ 0.001

Pesticide application induced an apparent reaction in the activity time of the bivalves in most cases. The most apparent reaction occurred in the case of DDVP. During the control period, the median activity time was 12 ± 3 h; after exposure it increased to 22 h. A very noticeable impact also was detected in the case of tebuconazole application. Here, during the control period, the median activity time was 17 ± 3 h, and after exposure, it decreased to 8 h (Fig. 5). Kruskal–Wallis ANOVA proved the high level of significance (rank tests) of daily activity time between the control and treatment period both for DDVP (*p* > 0.001, *H* = 46.23) and tebuconazole (*p* > 0.001, *H* = 37.61). A significant impact of DDT application was observed (*p* = 0.034, *H* = 18.49). In the case of lenacil and thiacloprid, a decrease in daily activity also was observed, but the differences between the control and treatment periods were not significant.

### Changes of Pesticides Concentration

**Fig. 6:**
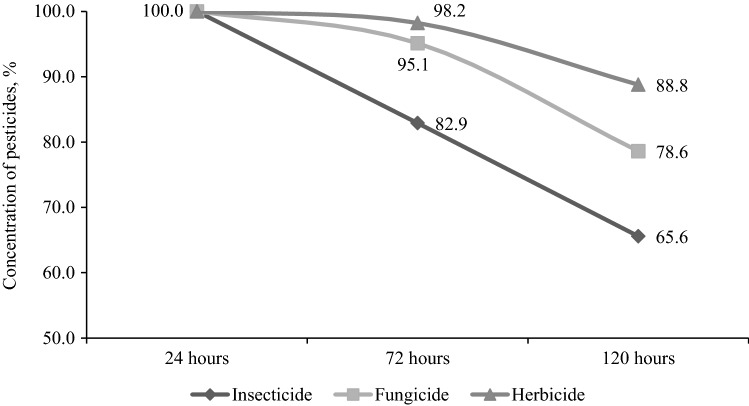
Pesticide concentration change during the experiment

During separate experiments, the variability of pesticide concentration was estimated. The concentration level decreased during the observation period (Fig. 6). The decrease in concentration was generally small, although some differences were observed between substances. The largest decrease in the residue concentration was recorded in case of the tested insecticides: between 24 and 120 h of exposure, the concentration decreased by 34.4%, compared with a decrease of 21.4% for the fungicide and only 11.2% for the herbicide (Fig. 6).

## Discussion

The results of the research show that the toxic effects of various pesticides on mussels is differentiated in terms of their impact on behaviour, including shell opening level, activity time, and shell opening rate, as well as the rapid onset of death. This study provides scientific evidence regarding the biological effects of pesticides on freshwater bivalves that are native to Poland. As reported by Renault (2011), the problem is serious, because contamination can have a negative impact on entire trophic chains of aquatic ecosystems.

Our analyses showed that pesticide contamination affects shell opening level to a small extent. Exposure to lenacil, DDT, thiacloprid, and tebuconazole has an insignificant effect on the reaction of *U. tumidus* (*p* > 0.05). Only bivalves exposed to DDVP showed significant change in shell opening level, which resulted from the death of all tested organisms. Therefore, the experiments demonstrated that even a high concentration of pesticides, under short-term exposure, has no influence on the shell opening level of the European unionoid *U.* *tumidus*. Bivalves usually keep their shells open more than 70% of the time (for oxygen and food intake). The total closure of shells occurs only if there is significant contamination of the water, because it involves significant energy losses (Kramer and Foekema 2001).

The study has shown that pesticide application affects shell opening rates. Exposure to lenacil, DDVP, thiacloprid, and tebuconazole had a highly significant effect on *U. tumidus* shell movement (*p* < 0.05). Only DDT treatment did not induce any behavioural reactions. The apparent change of shell opening rate under pesticide contamination has been reported in several previous studies (Ayad et al. 2011; Hartmann et al. 2016), but this pattern has a limited application in biomonitoring. Most biological warning systems are still based solely on the change in shell opening level (Bae and Park 2014). The introduction of shell opening rates into existing systems may increase the effectiveness of pesticide detection.

Although mortality during our short-term experiments was detected only in the case of DDVP contamination, all of the analysed substances have been demonstrated to have harmful effects in previous studies. Liess and Ohe (2005) reported that herbicides from surface runoff can be a cause of acute mortality in benthic invertebrates even at low concentrations. They also recorded a significant reduction in invertebrate taxonomic richness and abundance during spring, when the highest pesticide concentrations were found. Binelli et al. (2001) showed that DDTs have endocrine-disrupting effects in *Dreissena polymorpha* species. Oocyte degeneration may contribute to reductions in the population of individual species. In addition, most molluscs manifest a high accumulation of DDT in soft tissues. Binelli and Provini (2003) reported total DDT levels exceeding 3.12 μg g^−1^ in the soft tissues of *Dreissena polymorpha*. Moreover, DDT is still recorded in Poland, in both surface water and sediments. Reindl and Bolałek (2018) observed that in sediments of the Vistula Lagoon, concentrations of DDT lay within a range of 22.7–405.7 ng kg^−1^ dw. Several studies have investigated the effects of DDVP on living organisms, and in many cases this substance adversely affected bivalves. Relaxation of the adductor muscle of two bivalve species was reported by Le Bris et al. (1995). During 42 h of exposure to dichlorvos in concentrations of 0.1 and 1 μg L^−1^, bivalves behaved as if paralysed. Also, Bolton-Warberg et al. (2007) identified specific bivalve behaviour during a 96-h toxicity test. They observed relaxed adductor muscles in two species of molluscs subject to two DDVP concentrations (10 and 100 μg L^−1^). In the present study, we also observed similar bivalve behaviour under a DDVP concentration of 10 μg L^−1^. Based on the observations of Bolton-Warberg et al. this paralysis is caused by muscle fatigue. This may be a result of prolonged shrinkage of the muscles, associated with DDVP exposure. Neither Le Bris et al. (1995) nor Bolton-Warberg et al. (2007) observed any deaths.

In our study, all of the lethal cases were recorded after 7 days of exposure. Beketov and Liess (2008) observed delayed toxic effects occurring after exposure to thiacloprid. They admit that even with a 24-h exposure (at concentration 85 μg L^−1^, lower than the LC50 for *Daphnia magna*) death may be delayed. An increase in mortality was observed in several species 4–12 days after exposure. In our study, no fatalities were observed. At a concentration of 10 μg L^−1^, thiacloprid caused only an insignificant increase in the velocity of shell movement. Moreover, thiacloprid is included on the Watch List of contaminants of emerging concern (CECs) for European Union monitoring of surface water (launched in Decision 2015/495) (Sousa et al. 2019). Additionally, the effect of tebuconazole on freshwater invertebrates has been studied mainly for *Daphnia magna, Danio rerio*, *Gammarus fossarum*, etc. (Sancho et al. 2009; Zubrod et al. 2010; Andreu-Sánchez et al. 2012). However, Sancho et al. (2009) showed that the daily activity of daphnia decreased as tebuconazole concentration increased. The differences were observed after 96–120 h of exposure to 0.52 μg L^−1^ and higher concentrations of tebuconazole. In our study, we also observed reduced activity at a concentration of 10 μg L^−1^. Gammarids also showed significant reductions in activity, feeding, assimilation, and growth (Zubrod et al. 2010). The data suggest that tebuconazole is moderately toxic to invertebrates. However, it seriously impairs metabolic functions (Sancho et al. 2009; Andreu-Sánchez et al. 2012).

A decrease in the activity time, and thus the time of water filtration, was observed after exposure to lenacil, DDT, thiacloprid, and tebuconazole. However, statistically significant differences were demonstrated only for DDT and tebuconazole (*p* < 0.05). In the case of DDVP, the increase in activity resulted from muscle paralysis and thus the inability to close the shell. Even a small decrease in activity during the day reduces the amount of water filtered by the bivalves.

The great variability in terms of bivalves’ response depends on the duration of exposure, pesticide concentration or test species (Crestani et al. 2007; Modesto and Martinez 2010). However, Greco et al. (2010) concluded that other factors too, such as increased temperature, may modify bivalves’ response to pesticides. Aquatic creatures may be exposed to numerous variables. Abiotic factors (i.e., other pollutants, different temperatures, salinities, quantities of dissolved oxygen, and changes in pH) may accelerate the negative effect of pesticides (Broomhall 2002; Rohr et al. 2011).

Today, the tested pesticides are regularly detected and are recognised as a serious environmental threat (Beketov et al. 2013). According to the Water Framework Directive (European Commission 2000), all of the tested pesticides are regarded as toxic, and their active substances are included as priority substances (European Commission 2000). According to the Globally Harmonized System of Classification and Labelling of Chemicals (GHS), these pesticides are classified as H400: very toxic to aquatic life, or as H410: very toxic to aquatic life with long-lasting effects. All of them are regularly found in Poland and other European countries.

Two of the tested pesticides are not currently used (DDT and DDVP); however, their impact on the environment is still high, and the resulting risks are closely monitored. According to Directive 2013/39/EU, they are constant causes of mutagenic and carcinogenic fatal damage. Due to their particular ecological durability, both of them are still frequently detected in Poland (Kaczyński et al. 2017) and other parts of Europe (Oliveira et al. 2015; Quadroni and Bettinetti 2017; Lan et al. 2019), as well as outside Europe (Chen et al. 2016).

During the experiments, the nominal concentration 10 µg L^−1^ was used for each pesticide tested. Based on current European regulations, pesticide concentration in drinking water should not exceed 0.1 mg L^−1^ for a single pesticide (Council Directive 98/83/EC). However, in natural ecosystems the level of pesticides may be as much as 100 times higher (Satyavani et al. 2011).

Despite the fact that the impact of pesticides on ecosystems is under regular observation with the use of a wide range of scientific techniques, the use of bivalves has been shown to have considerable potential for water-quality monitoring. Based on behavioural observations, we have obtained a clear bioindicative response to water pollution by different types of pesticides. The investigations addressing the ecotoxicological effects of fungicides, herbicides, and insecticides on aquatic invertebrates seem to be adequate for a reliable assessment of adverse effects in freshwater ecosystems. However, further detailed studies are needed to utilize the bioindicative signals generated by bivalves under the stress of pollution and better understand the complex interactions between pesticides and the freshwater environment.
